# Performance metrics to unleash the power of self-driving labs in chemistry and materials science

**DOI:** 10.1038/s41467-024-45569-5

**Published:** 2024-02-14

**Authors:** Amanda A. Volk, Milad Abolhasani

**Affiliations:** Dept. of Chemical and Biomolecular Engineering, North Carolina State University, Raleigh, NC USA

**Keywords:** Chemical engineering, Automation, Techniques and instrumentation

## Abstract

With the rise of self-driving labs (SDLs) and automated experimentation across chemical and materials sciences, there is a considerable challenge in designing the best autonomous lab for a given problem based on published studies alone. Determining what digital and physical features are germane to a specific study is a critical aspect of SDL design that needs to be approached quantitatively. Even when controlling for features such as dimensionality, every experimental space has unique requirements and challenges that influence the design of the optimal physical platform and algorithm. Metrics such as optimization rate are therefore not necessarily indicative of the capabilities of an SDL across different studies. In this perspective, we highlight some of the critical metrics for quantifying performance in SDLs to better guide researchers in implementing the most suitable strategies. We then provide a brief review of the existing literature under the lens of quantified performance as well as heuristic recommendations for platform and experimental space pairings.

## Abstract (web summary)

Assessing the performance of self-driving labs (SDLs) is challenging due to the specificities of each experiment. In this Perspective, the authors review recent literature of SDLs for materials science and chemistry and suggest how to report SDL performance more effectively.

## Introduction

Self-driving labs (SDLs) are a rapidly growing field that offers incredible potential in improving the rate and scope of research in chemistry and materials science.^1^ SDLs are novel tools that incorporate automated experimental workflows (physical world) with algorithm-selected experimental parameters (digital world). Such autonomous experimentation tools can navigate complex and exponentially expanding reaction spaces with an efficiency unachievable through human-led manual experimentation, thereby allowing researchers to explore larger and more complicated experimental systems. At their highest degree of autonomy, the efficiency of SDLs can be derived from continuous, automated experimentation, which includes model retraining between each experiment. Such models can navigate and learn complex parameter spaces at a higher efficiency than the traditional design of experiment (DOE) approaches. These benefits thereby enable the discovery and optimization of novel and improved materials and molecules, as well as effective ways to manufacture them at scale. Due to the nascency of the SDL field in chemistry and materials science, the wide range of potential reaction space complexities, and the diversity of SDLs applied in literature, there is a need for system standards which define the criteria necessary for a system to qualify as autonomous or high performing. It should be noted that prior efforts have been made towards developing an SDL autonomy classification system for synthetic biology.^2,3^ In this article, building on the prior efforts of autonomy classification in synthetic biology,^2,3^ we propose a set of characterization metrics to delimitate between autonomy levels of SDLs in chemistry and materials sciences. Specifically, our proposed system explicitly defines the role of a human researcher for autonomy classification of SDL platforms in chemistry and materials science. While there is notable difficulty in directly comparing SDLs across different experimental spaces, many system features can be quantified and correlated meaningfully.

## Performance metrics for autonomous labs

**Fig. 1:**
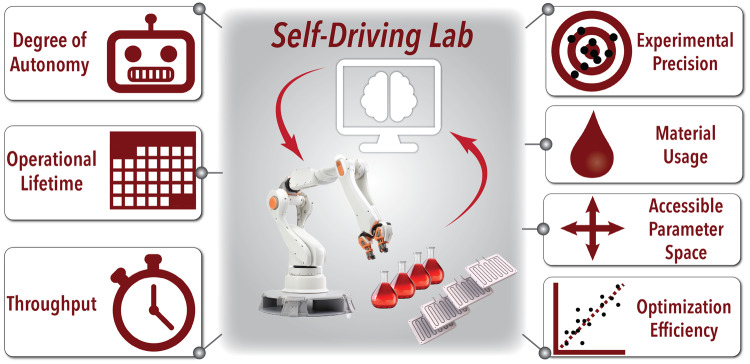
Key metrics for quantifying performance in SDLs. The metrics illustrated include degree of autonomy, operational lifetime, throughput, experimental precision, material usage, accessible parameter space, and optimization efficiency.

The features which can define the performance aspects of an SDL and are critical to report include specific information on the SDL’s degree of autonomy, operational lifetime, accessible parameter spaces, precision, throughput, sampling cost, and optimization performance – as shown in Fig. 1 and Table 1.

**Table 1:** Overview of the suggested performance metrics in SDLs with a summary for each metric, a list of reported studies that achieved a high degree of performance for each metric, and the subsequently reported metrics of the listed studies

*Metric*	*Summary*	*Exemplary Studies*	*Reported Metrics*
*Degree of Autonomy*	Classification of the extent with which human intervention is required for regular operation. The metric can be piecewise, semi-closed-loop, or closed-loop.	^4,13–16,18,20,23,26,29,30^	Closed-loop
*Operational Lifetime*	The total time that a platform can conduct experiments. The metric should be reported in four forms: demonstrated unassisted lifetime, demonstrated assisted lifetime, theoretical unassisted lifetime, and theoretical assisted lifetime. Additional research efforts should be made to evaluate the maximum lifetimes outside of case study optimizations.	^4^	700 samples (demonstrated, unassisted)
*Throughput*	The rate that the platform can conduct experiments. The metric should be reported in both demonstrated and theoretical throughput which includes both sample preparation and measurement. Additional research efforts should be made to evaluate the maximum throughput outside of case study optimizations.	^4,15^	30 to 33 samples per hr (demonstrated)
*Experimental Precision*	A quantitative value representing the reproducibility of an experimental platform. Precision estimates should be made using unbiased sequential experiments in conditions similar to those found during optimization. Sequential replication of a test condition can introduce bias.	^4,16,23^	Alternating random
*Material Usage*	The total quantity of materials used per experiment. The metric should be broken down into total active quantity during experimentation, total used per experiment, total hazardous material used per experiment, and total high value material used per experiment. The values should be reported in either volume or mass, where appropriate. Additional effort should be taken to include the material usage for auxiliary steps, such as reactor cleaning or preconditioning.	^4,13,24,25^	0.06 to 0.2 mL per sample
*Accessible Parameter Space*	Qualitative and quantitative description of accessible parameter space for a system along with the attainable measurement techniques. The reporting should be sub-divided into demonstrated and theoretical range.	^9^	1.6 × 10^11^
*Optimization Efficiency*	Quantitative analysis of the performance of a full system and its experiment selection algorithm. The most effective performance metric is direct algorithm benchmarking with replicates. The existing method can be compared with random sampling along with state-of-the-art selection algorithms. In the absence of sufficient data generation, simulated benchmarking can be applied. Where appropriate, linear regressions and explainable artificial intelligence techniques should be applied to any models used along with the required data set size to reach predictability.	^14–16,25^	Grid-search, SNOBFIT, CMA-ES, Nelder-Meade, and Human benchmarking

### Degree of autonomy

**Fig. 2:**
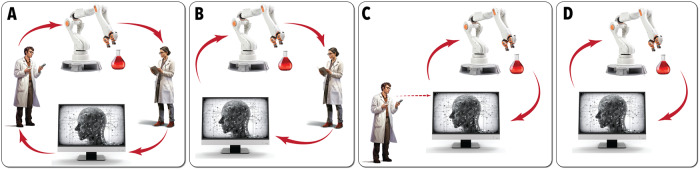
Degrees of autonomy in SDLs. Illustration of the process workflows for (**A**) piecewise, where human users fully separate the experiment and computational system, (**B**) semi-closed-loop, where the algorithm and robotic components partially communicate, (**C**) closed-loop, where the human user has no influence in the goal seeking loop, and (**D**) self-motivated experimental systems, where the computational system dictates its own objectives.

The degree of autonomy can be defined by the context in which non-robotic experimentalists may interact with the experimental system. Shown in Fig. 2, this feature may be broken down into piecewise, semi-closed loop, closed-loop, or self-motivated operation modules. A piecewise system, which may also be referred to as an algorithm-guided study, has complete separation between platform and algorithm. In this context, a human scientist must collect and transfer experimental data to the experimental selection algorithm. Once the algorithm picks the next experimental conditions, a human researcher must then transfer these to the physical platform to test. This piecewise schema is the simplest to achieve as there is no need for in/online or in-situ measurements, automated data analysis, or programming for robotics interfacing. These systems are particularly useful in informatics-based studies, high-cost experiments, and systems with low operational lifetimes since a human scientist can manually filter out erroneous conditions and correct system issues as they arise. However, this strategy is typically impractical for studies that require dense data spaces, such as high dimensional Bayesian optimization (BO) or reinforcement learning (RL). Next in degree of autonomy are semi-closed-loop systems. In these systems, a human scientist must interfere with some steps in the process loop, but there is still direct communication between the physical platform and the experiment-selection algorithm. Typically, the researcher must either collect measurements after the experiment or reset some aspect of the experimental system before experimental studies can continue. This technique is most applicable to batch or parallel processing of experimental conditions, studies that require detailed offline measurement techniques, and high complexity systems that cannot conduct experiments continuously in series. These systems are generally more efficient than a piecewise strategy while still accommodating measurement techniques that are not amenable to inline integration. However, they are often ineffective in generating very large data sets. Then, there are closed-loop systems, which further improves the degree of autonomy. A closed-loop system requires no human interference to carry out experiments. The entirety of the experimental conduction, system resetting, data collection and analysis, and experiment-selection, are carried out without any human intervention or interfacing. These systems are typically challenging to create; however, they offer extremely high data generation rates and enable otherwise inaccessible data-greedy algorithms (such as RL and BO). Finally, at the highest level of autonomy, will be self-motivated experimental systems which are able to define and pursue novel scientific objectives without user direction. These platforms merge the capabilities of closed-loop tools while achieving autonomous identification of novel synthetic goals, thereby removing the influence of a human researcher. No platform to date has achieved this level of autonomy, but it represents the complete replacement of human guided scientific discovery.

### Operational lifetime

In conjunction with the degree of autonomy, it is also important to consider the operational lifetime of an SDL. Quantification of this value enables researchers to understand when platforms are suited to their data, labor, and platform generation budgets. Operational lifetime can be divided into four categories: demonstrated unassisted lifetime, demonstrated assisted lifetime, theoretical unassisted lifetime, and theoretical assisted lifetime. The distinction between theoretical and demonstrated lifetimes allows researchers to showcase the full potential of an SDL without misrepresenting the work that was carried out. For example, the operational lifetime of a microfluidic reactor is constrained to the volume of source chemicals provided as well as additional factors such as precursor degradation or reactor fouling. In practice, most microfluidic studies feature demonstrated lifetimes on the scale of hours. However, without source chemical limitations, many of these systems may reach functionally indefinite theoretical lifetimes. Even with these theoretical indefinite lifetimes, reporting demonstrated lifetimes and their context is critical to communicating the potential application of a platform. For example, demonstrated lifetime should be specified as the maximum achieved lifetime or, more importantly, the average demonstrated lifetime across trials. In addition, assisted and unassisted demonstrated lifetimes should be clarified to help identify labor requirements and therefore scalability of an SDL. For example, in recent work by the authors, a microdroplet reactor was used to conduct colloidal atomic layer deposition reactions over multiple cycles.^4^ One precursor used would degrade within two days of synthesis, and a fresh precursor was needed to be prepared once every two days. Beyond this limitation, the SDL could run continuously for one month without stopping or needing to be cleaned. In this study, the demonstrated unassisted lifetime is two days, and the demonstrated assisted lifetime is up to one month.

### Throughput

Like operational lifetime, throughput is a critical component in specifying the capability of an automated system. Throughput is often referenced as the primary metric with which to compare technologies, as it is the most common bottleneck in achieving dense data spaces. As such, many techniques and fields distinguish themselves through this metric. However, throughput is often heavily dependent on the experimental system being studied as well as the technique being used to measure the material. For example, a platform can be highly efficient in conducting experiments, but if it is studying a synthesis with a long reaction time and does not have parallelization capability, the throughput is significantly throttled. Alternatively, if an experimental space includes a rapid reaction time, but the characterization method is too slow to sufficiently capture early time scales, then a large portion of the parameter space is neglected. Furthermore, if a characterization method is non-destructive, a single sample can generate multiple measurements, thereby enabling a significantly higher data generation rate. Consequently, the throughput is best reported as both theoretical and demonstrated values, which encompasses both the platform material preparation rate and the analyses. As an example, from work published by the authors, in a microfluidic rapid spectral sampling system presented previously, the platform could generate over 1,200 measurements per hour while running at maximum throughput, but for the longer reaction times studied, the actual sampling rate was closer to 100 measurements per hour.^4^ Therefore, this work showed a demonstrated throughput of 100 samples per hour and a theoretical throughput of 1,200 measurements per hour. The combination of these two values provides context on both the maximum potential limit and the actual stress tested limit.

### Experimental precision

**Fig. 3:**
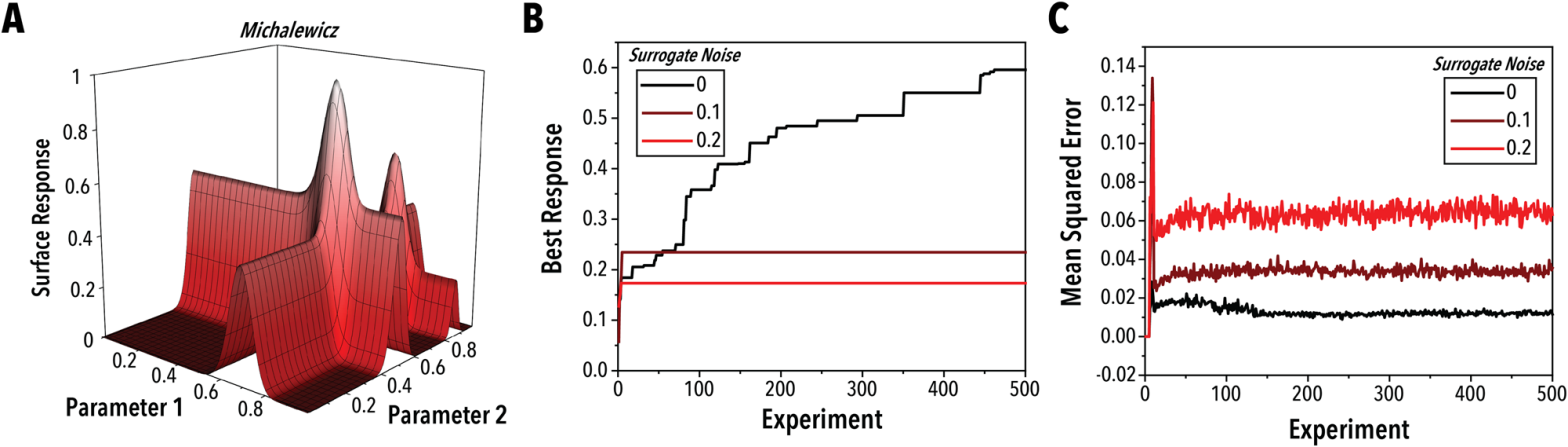
Effect of noise on optimization efficiency. **A** Surface response plot of a two-dimensional michalewicz surrogate function, (**B**) median best response and (**C**) median mean squared error across ten replicates for a simulated optimization of a six-dimensional michalewicz surface with varying degrees of noise indicated by the legend. As the level of noise observed in the surrogate function is increased, the performance of the optimization algorithm decreases while the algorithm model’s uncertainty increases. More precise experimental platforms, therefore, tend to generate higher performing self-driving laboratories. The optimization algorithm uses bagging regression with an exhaustive grid search hyperparameter tuned multi-layered perceptron and an upper confidence bounds decision policy. Noise is applied to the surrogate function by randomly sampling from a normal probability function with standard deviations of 0, 0.1, and 0.2 respectively and adding the sample to the surrogate output.

Experimental precision represents the unavoidable spread of data points around a “ground truth” mean value. Precision can be quantified by the standard deviation of replicates of a single condition, conducted in an unbiased manner. Recently, there has been increased focus on the significance of this metric in SDLs, particularly through the use of simulated experimentation through surrogate benchmarking. Surrogate benchmarking is used to evaluate algorithm performance on different parameter spaces without requiring operation of a full experimental system. Instead of conducting physical experiments, the algorithm samples from a simple function digitally, thereby significantly increasing the throughput and offering direct comparisons between algorithms through the evaluation of standardized, n-dimensional functions.^5–8^ Shown in Fig. 3, sampling precision has a significant impact on the rate at which a black-box optimization algorithm can navigate a parameter space,^5,9,10^ a finding that is supported by prior literature.^11^ In many cases, high data generation throughput cannot compensate for the effects of imprecise experiment conduction and sampling. Therefore, it is critical to develop SDL hardware that can generate both large and precise data sets. Characterization of the precision component is, therefore, critical for evaluating the efficacy of an experimental system. The ideal protocol for acquiring this metric is to conduct unbiased replicates of a single experimental condition set. There are many ways to conduct these replicates, and the exact methods for preventing bias will vary from system to system. However, the most common bias to avoid is through sequential sampling of the same conditions. As shown in prior literature, the test condition can be alternated with a random condition set before each replicate. This sampling strategy helps to position the test condition in an environment more similar to the setting used for optimization.

### Material usage

When working with the number of experiments necessary for algorithm-guided research and navigation of large, complex parameter spaces, the quantity of materials used in each trial becomes a consideration. This consideration can be broken down into safety, monetary costs, and environmental impacts. Lower working volumes of hazardous materials in a platform means that critical failures can be more easily contained, which expands the parameter space of exploration to unforeseen results and a larger library of reaction candidates. Therefore, it is important to report the total active quantity of particularly hazardous materials. Furthermore, low material usage reduces the overall cost and environmental impacts of experimentation. For research involving expensive or environmentally harmful materials, it is important to quantify the impacts of the reaction system. As such, experimental costs should be reported in terms of usage of the total materials, high value materials, and environmentally hazardous materials. Total material and environmentally hazardous material generation should be reported with respect to the total quantities used, which includes waste stream materials generated through system washing and measurement references. It should be noted that many processes developed with microscale experimental systems are difficult to scale to functional quantities. Therefore, where applicable, it is important to provide data quantifying the scalability or generated knowledge of a developed process.

### Accessible parameter space

Beyond the baseline characteristics associated with the quantity and quality of the data generated, another important consideration is the possible range of experimental parameters that can be accessed on both the inputs and outputs. Every experiment conduction strategy features its own limitations on the accessible parameter space, and each poses further limitations by the tools used to measure them. Liquid handling robots typically are limited from handling extremely low reaction times, and microfluidic reactors typically require solution phase precursors and are constrained to by injection ratios. Precise reporting of the demonstrated and theoretical parameter space along with details of the characterization techniques is critical for communicating the capabilities and limitations of an SDL. Each of the parameters used in a study should be reported alongside their minimum and maximum bounds and how they are parameterized in the optimization algorithms. Furthermore, considerable effort should be made to include qualitative constraints on the accessible list of parameters that may be used by an SDL.

### Optimization efficiency

**Fig. 4:**
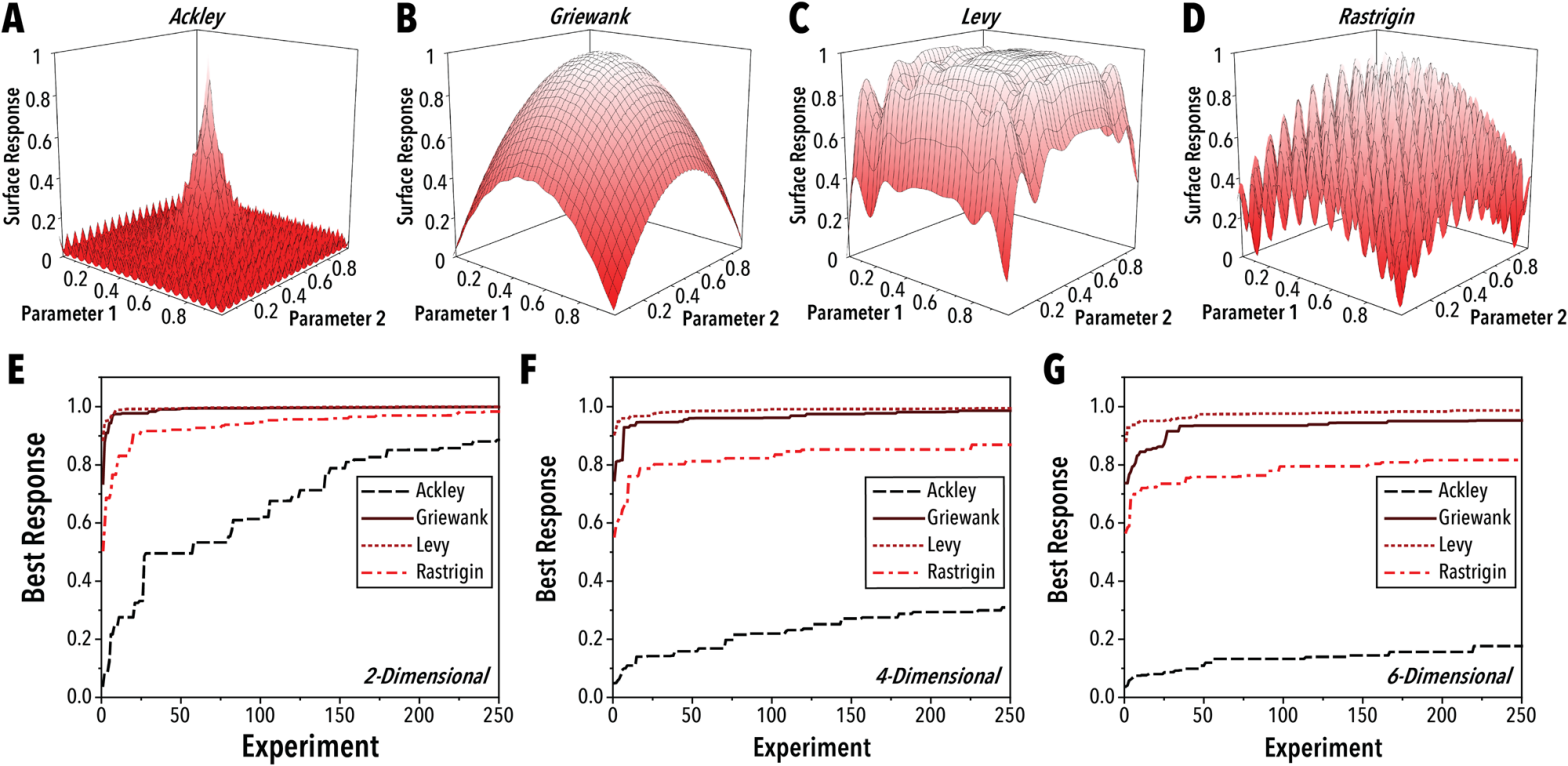
Effect of surface complexity on optimization rate. Two-dimensional surface plots of the surrogate functions (**A**) Ackley, (**B**) Griewank, (**C**) Levy, and (**D**) Rastrigin and the median best response of ten optimization replicates across the four surrogates in (**E**) two-, (**F**) four-, and (**G**) six-dimensional parameter spaces. The optimization algorithm consists of gaussian processor regression with an upper confidence bounds decision policy.

Finally, and likely most importantly, every SDL study should include a comprehensive evaluation of the overall system performance. Benchmarking with a real-world, experimental platform can be highly challenging, as there is often little data available for direct comparison, and it is typically too costly to conduct replicates with alternative systems or algorithms. Moreover, two seemingly similar experimental systems can feature reaction spaces of differing complexity, resulting in a more challenging optimization for one than the other. Shown in Fig. 4, many aspects of surface response features can influence the rate of optimization. With these limitations in mind, there are several aspects of a physical platform and the experiment-selection algorithm of SDLs that can serve as reasonable indicators of their performance. First, it is important to specify the optimized feature that was achieved because of the study along with the number of experiments or prior data implemented to reach that outcome. Where relevant, all champion results should be benchmarked with appropriate state-of-the-art literature. Next, the algorithm should be demonstrated to provide basic predictability across the studied data set. In model-driven algorithms, this can be provided through a simple regression validation by splitting all the available data into training and testing sets and predicting the outcome of unknown measurements. Furthermore, there should be a clear discussion of the dimensionality of the parameter space explored along with quantification of each parameter’s degree of influence. With increasing interest in explainable AI, there are libraries of simple tools, such as Shapley plots, for quantifying the influence of each parameter on the system response.^12^ With model-driven algorithms, extracting these values is as simple as running the model through a prebuilt algorithm. Finally, when there are no apparent benchmarks for a given experimental space, random sampling can serve as a simple and clear standard. By comparing the performance of an experiment-selection algorithm to random sampling, the researcher can demonstrate control over the experimental space. Outside of serendipitous trials, the only way to achieve an experiment-selection algorithm that bypasses the performance of randomly selected conditions is to build a functioning autonomous platform with an effective guiding algorithm.

## Self-driving laboratories in literature

**Fig. 5:**
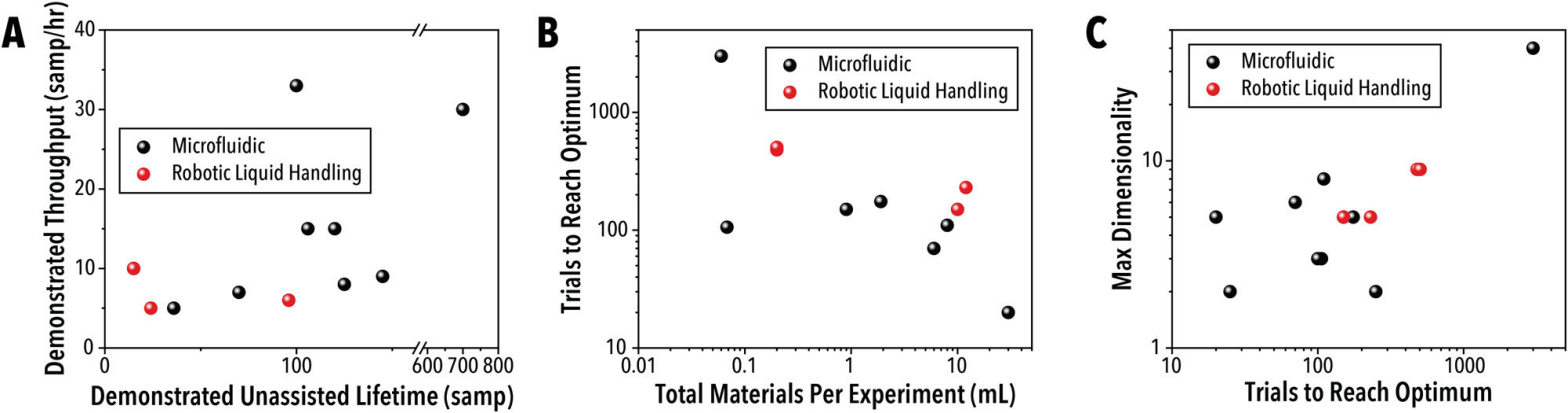
Analysis of SDL Performance in Literature. **A** The system throughput as a function of demonstrated unassisted lifetime, **B** the number of trials required to reach the optimum value as a function of the total material cost per experiment, and **C** the dimensionality of the parameter space as a function of the number of trials required to reach the optimum for both liquid handler and microfluidics based automated systems. Note that publications that do not report the listed values are not included in the figure.

By clearly reporting the parameters detailed in this perspective, research can be guided towards more productive and promising technological areas. Early evaluation of these metrics under a sampling of recent SDL literature – detailed in Table 1 – leads to several technological indicators that can already affect decision-making in SDL studies.^4,13–28^ First, of the available technologies, microfluidic platforms have demonstrated unassisted generation of larger data sets and at a higher demonstrated throughput, as shown in Fig. 5. Among liquid handling tools, micro-well plate systems were at the top in performance. Second, there is a slight correlation between experimental cost and the total number of trials used to reach the optimum condition. Experimental systems that consume small quantities of materials can generate larger data sets and, therefore, apply more resources toward process optimization. Both indicators suggest that low material consumption technologies are the most effective in black-box optimization environments in the current state of SDL technology. However, these points should be taken with a major caveat. In much of the SDL literature mining performed for this perspective, data generation rates are largely limited by the reaction rates under study. Few SDL papers report system specifications beyond what is necessary for a case study experiment, but in studies that present an SDL as the core of the work, these parameters are just as important as the exact experiments that are conducted. Improved reporting and stress testing of SDLs would help to resolve this deficiency in the available data and direct further research into more effective and productive technologies.

Additionally, the sampled SDL literature, shown in supporting information Table S.1, does not show a clear correlation between the dimensionality of the studied parameter space and the number of trials required to reach an optimum. Some deviation in the required number of trials is expected, due to varying complexity of the response surfaces and the presence of non-contributing parameters. However, a correlation with dimensionality should be present, particularly when assuming real-world experimental systems tend to exhibit similar levels of complexity. This trend indicates that many of the prior works do not provide the global optimum of the studied experimental space. This is to be expected, as identifying when a global optimum has been reached is a fundamental and largely unsolvable challenge in the optimization of high-cost experimental spaces. With no clear, quantifiable indicator of a comprehensively explored and optimized space available, alternative metrics for demonstrating an SDL efficacy are necessary.

As previously discussed, it is critical to report SDL’s algorithm performance features in formats that demonstrate predictive capabilities, feature analyses, and benchmarking, yet these parameters are not often included in the SDL literature. Among the seventeen surveyed studies shown in supporting information Table S.1, 23% included a real-world benchmarking of any kind, and 12% included simulated benchmarking, leaving 65% of the studies without any form of algorithm comparison. Additionally, only 62% of the thirteen studies that leverage a machine learning model demonstrated any form of model validation, and only 19% conducted any parameter analysis. Furthermore, 71% of the studies reported no data quantifying the precision of the automated experimental system of the built SDL. Finally, no quantitative information on the accessible parameter space was found in the selection of reported literature. With this absence of information on the basic performance metrics of SDLs, it is highly challenging to elucidate a clear direction for the field. A larger effort should be taken by researchers to ensure that these quantitative metrics are included.

## Conclusions

It is critical to the development of future SDLs that studies include clear and precise efforts to quantify the capabilities of the presented platform. Without more deliberate and thorough evaluation of SDLs, the field will lack the necessary information for guiding future research. However, due to the inherently different challenges posed by each experimental space, there is a significant difficulty in comparing performance between systems by features such as optimization rate. Additionally, there is not a clear indicator to identify a fully optimized experimental space in high experimental cost problems. Instead, it is more effective to apply the criteria laid out in this perspective and include quantified data regarding the performance of the platform, software, and combined system. By doing so, the knowledge gap in the existing SDL literature will be better filled, and researchers can pursue quantifiably promising research directions.

### Supplementary information

Supplementary Information

## Data Availability

The source data generated in this study have been deposited in the repository “SDL” (https://github.com/AbolhasaniLab/SDL).

## Code availability

The source code for the noise benchmarking plots and surrogate models have been deposited in the repository “SDL” (https://github.com/AbolhasaniLab/SDL).
