# Supplementary material for: Performance metrics to unleash the power of self-driving labs in chemistry and materials science
Source: Nat Commun. 2024 Feb 14;15:1378. doi: 10.1038/s41467-024-45569-5 (PMC10866889; doi:10.1038/s41467-024-45569-5)
Supplement: Supplementary file 1 — Supplementary Information [file 41467_2024_45569_MOESM1_ESM.pdf]

Supporting Information

Performance Metrics to Unleash the Power of Self-Driving Labs in Chemistry and Materials Science

Amanda A. Volk and Milad Abolhasani*

Dept. of Chemical and Biomolecular Engineering, North Carolina State University, Raleigh, NC, USA

E-mail: abolhasani@ncsu.edu

Table S.1 - Summary of existing benchmarking efforts found from a sampling of the literature.

**Base**	***Reference***	1	2	3	4	5	6	7	8	9	10	11	12	13	14	15	16	17
	Field of Research	Nanoparticles	Nanoparticles	Nanoparticles	Nanoparticles	Nanoparticles	Nanoparticles	Nanoparticles	Nanoparticles	Nanoparticles	Nanoparticles	Bose Einstein condesates	RAFT Polymer	Short Chain Polymers	Polymer-Protein Hyrids	Medical Materials	Molecular Beam Epitaxy	Molecular Beam Epitaxy
	Material Studied	CdSe QDs	(Cs/FA)Pb(I/Br)$_3$ QDs, (Rb/Cs/FA)Pb(I/Br)$_3$ QDs	Chiral CsPbBr3 QDs	Au NP	CsPbBr$_3$ QDs	CsPbX$_3$ QDs	Ag NP	Mn:CsPb(Cl/Br)$_3$	Au NPs	CdSe/CdS	ultra-cold-atoms	P(tBuAm) RAFT agents	Short Polymer Fiber	horseradish peroxidase, glucose oxidase	Chondroitinase ABC with Polymer	SrRuO$_3$	TiN
	Max Dimensionality	3	3	2	5	5	8	5	6	5	40	16	2	5	9	9	3	4
	Algorithm	SNOBFIT	Bayesian Optimization - Gaussian Processes	SNOBFIT	Evolutionary Algorithm	Bayesian Optimization - Ensemble Neural Networks	Bayesian Optimization - Ensemble Neural Networks	Bayesian Optimization - Gaussian Processes	Bayesian Optimization - Ensemble Neural Networks	Evolutionary Algorithm	Reinforcement Learning	Bayesian Optimization - Gaussian Processes	TSEMO	Bayesian Optimization - Gaussian processes	Bayesian Optimization -Gaussian Processes	Bayesian Optimization -Gaussian Processes	Bayesian Optimization - Gaussian Processes	Bayesian Optimization - Gaussian Processes
	Experimental Platform	Microfluidic	Microfluidic	Microfluidic / Robotic	Robotic Liquid Handling	Microfluidic	Microfluidic	Microfluidic	Microfluidic	Robotic Liquid Handling	Microfluidic	Atom laser	Microfluidic	Microfluidic	Robotic Liquid Handling	Robotic Liquid Handling	molecular beam epitaxy	Robotic Molecular Beam Epitaxy
	Degree of Autonomy	Closed-loop	Closed-loop	Closed-loop	Semi-Closed-loop	Closed-loop	Closed-loop	Semi-Closed-loop	Closed-loop	Semi-Closed-loop	Closed-loop	Closed-loop	Closed-loop	Piecewise	Piecewise	Piecewise	Piecewise	Piecewise
**Lifetime**	***Demonstrated Unassisted Lifetime (samp)***	*106*	100	120	15	145	125	15	70	24	700	145	36	1	96	96	1	1
	Demonstrated Assisted Lifetime	*636*	220	250	150	1400	250	150	110	1000	9000	410	140	1	480	504	30	20
	Theoretical Unassisted Lifetime	*Indef.*	*Indef.*	-	15	*Indef.*	*Indef.*	*Indef.*	*Indef.*	*24*	*Indef.*	Indef.	-	-	96	96	-	-
	Theoretical Assisted Lifetime	*Indef.*	*Indef.*	-	*Indef.*	*Indef.*	*Indef.*	*Indef.*	*Indef.*	*Indef.*	*Indef.*	Indef.	-	-	Indef.	Indef.	-	-
**Throughput**	***Demonstrated Throughput (samp/hr)***	*15*	33	15	10	9	8	10	7	5	30	-	5	-	6	6	-	-
	Theoretical Throughput	-	-	-	-	-	-	-	-	10	-	-	-	-	-	-	-	-
**Precision**	***Precision Assessment Method***	-	Continuous Sampling	-	-	Alternating Random, Continuous Sampling	-	-	Alternating Random	Continuous Sampling	Alternating Random, Continuous Sampling	-	-	-	-	-	-	-
**Quantity**	***Maximum Active Quantity***	0.017 mL	-	-	150 mL	0.5 mL	0.8 mL	-	1.5 mL	288 mL	0.015 mL	N/A	2 mL	-	20 mL	20 mL	N/A	N/A
	Total Materials Per Experiment	0.068 mL	-	-	10 mL	1.9 mL	8 mL	0.9 mL	6 mL	12 mL	0.06 mL	N/A	-	30	0.2 mL	0.2 mL	N/A	N/A
	Total Hazardous Per Experiment	0.068 mL	-	-	N/A	1.4 mL	4 mL	0.9 mL	6 mL	12 mL	0.01 mL	N/A	N/A	N/A	N/A	N/A	N/A	N/A
	Total High Value Per Experiment	N/A	N/A	N/A	N/A	N/A	N/A	N/A	N/A	N/A	N/A	N/A	N/A	N/A	N/A	N/A	N/A	N/A
**Algorithm Performance**	***Trials to Reach Optimum***	106	100	250	150	175	110	150	70	230	3000	35	25	20	480	504	30	20
	Model Validation	N/A	Regression	N/A	N/A	Mean Squared Error	-	Regression, Mean Squared Error	Regression	N/A	Regression	-	-	-	Cross Validation	Cross Validation, Mean squared error	-	Regression
	Feature Analysis	-	-	-	-	-	-	Shapley	Shapley	-	N/A	-	-	-	Shapley	-	-	-
	Benchmarking	-	Grid Search	-	-	SNOBFIT, CMA-ES	-	-	-	Random (Simulation)	Bayesian Optimization - Gaussian Processes (Simulation)	Nelder-Meade	-	-	Human Guided	-	-	-

Supplementary References

1 S. Krishnadasan, R. J. C. Brown, A. J. DeMello and J. C. DeMello, *Lab Chip*, 2007, **7**, 1434–1441.

2 L. Bezinge, R. M. Maceiczyk, I. Lignos, M. V. Kovalenko and A. J. deMello, *ACS Appl. Mater. Interfaces*, 2018, **10**, 18869–18878.

3 J. J. J. Li, R. Liu, Y. Tu, Y. Li, J. Cheng, T. He and X. Zhu, *Nat. Commun.*, 2020, **11**, 1–10.

4 D. Salley, G. Keenan, J. Grizou, A. Sharma, S. Martín and L. Cronin, *Nat. Commun.*, 2020, **11**, 1–7.

5 R. W. Epps, M. S. Bowen, A. A. Volk, K. Abdel-Latif, S. Han, K. G. Reyes, A. Amassian and M. Abolhasani, *Adv. Mater.*, 2020, **32**, 2001626.

6 K. Abdel-Latif, R. W. Epps, F. Bateni, S. Han, K. G. Reyes and M. Abolhasani, *Adv. Intell. Syst.*, 2021, **3**, 2000245.

7 F. Mekki-Berrada, Z. Ren, T. Huang, W. K. Wong, F. Zheng, J. Xie, I. P. S. Tian, S. Jayavelu, Z. Mahfoud, D. Bash, K. Hippalgaonkar, S. Khan, T. Buonassisi, Q. Li and X. Wang, *npj Comput. Mater.*, 2020, **7**, 1–10.

8 F. Bateni, R. W. Epps, K. Antami, R. Dargis, J. A. Bennett, K. G. Reyes and M. Abolhasani, *Adv. Intell. Syst.*, 2022, **4**, 2200017.

9 Y. Jiang, D. Salley, A. Sharma, G. Keenan, M. Mullin and L. Cronin, *Sci. Adv.*, 2022, **8**, abo2626.

10 A. A. Volk, R. W. Epps, D. T. Yonemoto, B. S. Masters, F. N. Castellano, K. G. Reyes and M. Abolhasani, *Nat. Commun.*, 2023, **14**, 1–16.

11 P. B. Wigley, P. J. Everitt, A. Van Den Hengel, J. W. Bastian, M. A. Sooriyabandara, G. D. Mcdonald, K. S. Hardman, C. D. Quinlivan, P. Manju, C. C. N. Kuhn, I. R. Petersen, A. N. Luiten, J. J. Hope, N. P. Robins and M. R. Hush, *Sci. Rep.*, 2016, **6**, 1–6.

12 S. T. Knox, S. J. Parkinson, C. Y. P. Wilding, R. A. Bourne and N. J. Warren, *Polym. Chem.*, 2022, **13**, 1576–1585.

13 C. Li, D. Rubín De Celis Leal, S. Rana, S. Gupta, A. Sutti, S. Greenhill, T. Slezak, M. Height and S. Venkatesh, *Sci. Rep.*, 2017, **7**, 1–10.

14 M. J. Tamasi, R. A. Patel, C. H. Borca, S. Kosuri, H. Mugnier, R. Upadhya, N. S. Murthy, M. A. Webb and A. J. Gormley, *Adv. Mater.*, 2022, **34**, 2201809.

15 S. Kosuri, C. H. Borca, H. Mugnier, M. Tamasi, R. A. Patel, I. Perez, S. Kumar, Z. Finkel, R. Schloss, L. Cai, M. L. Yarmush, M. A. Webb and A. J. Gormley, *Adv. Healthc. Mater.*, 2022, **11**, 2102101.

16 Y. K. Wakabayashi, T. Otsuka, Y. Krockenberger, H. Sawada, Y. Taniyasu and H. Yamamoto, *APL Mater.*, 2019, **7**, 101114.

17 I. Ohkubo, Z. Hou, J. N. Lee, T. Aizawa, M. Lippmaa, T. Chikyow, K. Tsuda and T. Mori, *Mater. Today Phys.*, 2021, **16**, 100296.
